# Direct and indirect effects of alexithymia on disordered eating in a non-clinical female sample: Determining the role of negative affect

**DOI:** 10.3389/fpsyt.2022.994024

**Published:** 2022-11-24

**Authors:** Deborah J. Wallis, Nathan Ridout

**Affiliations:** ^1^Faculty of Health and Life Sciences, School of Applied Social Sciences, De Montfort University, Leicester, United Kingdom; ^2^School of Psychology, College of Health and Life Sciences, Aston University, Birmingham, United Kingdom

**Keywords:** emotional awareness, affect, eating disorders, depression, anxiety, mediation

## Abstract

**Background:**

Alexithymia is an independent predictor of symptoms of eating disorders, but also influences disordered eating in clinical samples indirectly *via* negative affect (depression and anxiety). The aim of the current work was to establish if alexithymia predicts disordered eating in a non-clinical sample directly and indirectly (*via* negative affect).

**Methods:**

A sample of healthy females (*n* = 248) completed measures of depression, anxiety, alexithymia, and disordered eating (*drive for thinness*, *bulimia*, and *body dissatisfaction*). Bias-corrected bootstrapping was used to conduct parallel mediation analyses to determine if negative affect (depression and anxiety) mediated the influence of alexithymia on disordered eating.

**Results:**

The relationship between alexithymia (*difficulty identifying feelings)* and *drive for thinness* was mediated by depression but not anxiety. The link between *difficulty identifying feelings* and *bulimia* was mediated by anxiety but not depression. The correlation between alexithymia (*difficulty describing feelings*) and *body dissatisfaction* was mediated by both depression and anxiety. However, after controlling for negative affect, *difficulty identifying feelings* remained an independent predictor of *drive for thinness*, and *difficulty describing feelings* remained an independent predictor of *body dissatisfaction.*

**Conclusion:**

Facets of alexithymia (DIF and DDF) directly predict disordered eating in healthy participants as well as indirectly *via* depression and anxiety. These findings suggest that targeted interventions to improve the ability of individuals to identify and describe their feelings could be beneficial in reducing disordered eating, particularly in those “at risk” of developing eating disorders.

## Introduction

Emotion regulation deficits are a core feature of eating disorders: anorexia nervosa (1), bulimia nervosa (2), and binge eating disorder (3). An important factor thought to underlie emotion dysregulation in individuals with eating disorders is alexithymia (4). Alexithymia (ALX) is a multi-faceted construct characterised by problems identifying one’s feelings, distinguishing between feelings and bodily sensations, difficulties in verbally describing one’s feelings to others, a lack of imagination and fantasies, and an externally focussed cognitive style (5). The most widely used measure of alexithymia is the 20-item Toronto Alexithymia Scale [TAS-20 (6, 7)], which consists of three subscales: *difficulties identifying feelings* (DIF), *difficulties describing feelings* (DIF), and *externally-oriented thinking* (EOT).

Studies using the TAS-20 have consistently shown high levels of alexithymia in patients with eating disorders: anorexia nervosa (8, 9), bulimia nervosa (10, 11), and binge eating disorder (12, 13), see also reviews (4, 14). Furthermore, direct links have been reported between scores on the alexithymia subscales DIF and DDF, and symptoms in patients with eating disorders (9, 12, 15, 16), which suggests that these facets might play a role in the development of disordered eating. In line with this proposal, longitudinal evidence revealed that alexithymia (scores on the DIF subscale) is a negative prognostic factor for outcomes in patients with eating disorders (17).

It has been suggested that alexithymia might be an enduring characteristic of individuals who are liable to develop disordered eating. Evidence in support of this proposal comes from studies reporting no change in alexithymia levels in patients in remission from their eating disorder (18, 19). However, other findings have revealed significant reductions in alexithymia as eating disorder symptoms improve (15, 20), which suggests that alexithymia might be a state feature of disordered eating rather than a dispositional influence. Nevertheless, even in studies that show reduced alexithymia scores upon remission, individuals who have experienced eating disorders still tend to report significantly higher alexithymia scores than do healthy controls with no history of disordered eating (10, 15, 16). Taken together, evidence supports the conception of alexithymia as a relatively stable feature of individuals who are at risk of developing disordered eating. This is also consistent with evidence of relative stability of alexithymia in patients with depression (21, 22), anxiety disorders (23), and in the general population (24, 25).

An important issue is that many aspects of disordered eating are dimensional not only across eating disorder diagnoses but also across the general population. For example, taxometric analysis has revealed that that dietary restraint, body dissatisfaction, and drive for thinness are dimensional across clinical and non-clinical samples (26). Furthermore, there is also evidence of a similar dimensional nature to binge eating (27). Given the conception of alexithymia as a trait vulnerability factor for disordered eating, relationships between alexithymia and measures of disordered eating would be expected within non-clinical samples. In line with this expectation, positive associations have been observed between total TAS-20 score and total score on the Eating Disorders Inventory [EDI (28)] in a sample of healthy participants (29). Positive correlations have also been found between the full TAS-20 score and the total score on the Eating Attitudes Test [EAT-26 (30)], and scores on all three subscales of the EAT-26: *dieting*, *bulimia*, and *oral control* (31). In another study the observed relationships between full TAS-20 scores and EAT-26 scores were limited to the total EAT-26 score and score on the *bulimia* subscale (32). The link between alexithymia (total TAS-20 score) and bulimic behaviours has been confirmed in another non-clinical sample (33). Studies examining the influence of the different facets of alexithymia in healthy participants have revealed that DIF and DDF scores are positively related to scores on the *drive for thinness*, *bulimia*, and *body dissatisfaction* scores on the EDI (34) and to scores on all three subscales of the EAT-26 (35). However, in other work using the EDI scores on the DIF and DDF subscales were only related to scores on the *bulimia* subscale (36). Taken together there is clear evidence that alexithymia is associated with disordered eating in non-clinical samples, but the nature of this relationship is not stable across studies. Thus, there is a need to examine factors that might influence the relationship between alexithymia and disordered eating.

One potentially important factor is negative affect, particularly depression and anxiety. High levels of alexithymia have been observed in individuals with depression (21, 37–40) and anxiety (23, 38). Furthermore, there is evidence that depression and anxiety are risk factors for the development of eating disorders (41–45). With this in mind, an important theoretical issue concerns whether the link between alexithymia and disordered eating can be explained by comorbid negative affect (depression and/or anxiety). A meta-analysis of the rates of alexithymia in clinically diagnosed eating disorders reported that, in 11 of the 19 studies that had controlled for depression (some also controlled for anxiety), differences in alexithymia between patients with eating disorders and healthy controls disappeared once the influence of negative affect had been controlled (14). When considering the non-clinical data, only three of the seven studies reporting significant relationships between alexithymia and disordered eating had controlled for negative affect. However, two of those that did control for depression reported that relationships between alexithymia and disordered eating remained after controlling for negative affect (29, 33) with only one showing that negative affect fully explained the link between alexithymia and disordered eating (34). One possibility is that negative affect (depression and anxiety) might mediate the effect of alexithymia on disordered eating. That is, alexithymia might influence disordered eating indirectly *via* negative affect.

One study that examined this proposal used mediation analysis to determine the direct and indirect effects of alexithymia on symptoms of anorexia nervosa, *via* the mediator depression (46). The direct effect refers to the relationship between alexithymia and anorexia, controlling for the influence of depression. The indirect effect refers to the influence of alexithymia on anorexia that occurs *via* depression; that is, alexithymia is linked to high levels of depression and high levels of depression are linked to more severe symptoms of anorexia. The results of the mediation analysis revealed evidence of both direct and indirect effects of alexithymia on anorexia (46). Another study examined the direct and indirect effects of alexithymia on disordered eating (measured by the EAT-26) *via* dual mediators of depression and anxiety within a sample of patients with borderline personality disorder (47). The results of this study showed that both depression and anxiety mediated the influence of alexithymia on disordered eating symptoms (indirect pathway). However, even after controlling for the influence of negative affect, there was still evidence of a direct effect of alexithymia on disordered eating. Taken together these studies suggest that alexithymia in clinical populations is an independent predictor of disordered eating, in addition to an indirect influence *via* negative affect.

It is important to examine if these relationships hold in a subclinical samples, as rates of subthreshold disordered eating are higher than those of clinically diagnosed eating disorders (48). For example, a recent study conducted in a cohort of Australian adolescents revealed that around 25% of the sample exhibited subclinical levels of disordered eating (49). This is notable as subclinical disordered eating within the general population has been shown to have significant negative effects on quality of life (50). No study has yet directly examined if negative affect mediates the influence of alexithymia on disordered within a non-clinical population. However, one study conducted two separate mediation analyses and demonstrated that depression and anxiety mediated the effect of alexithymia on emotional eating. This suggests there is utility in conducting mediation analyses to understand the complex relationships between alexithymia, mood, and eating behaviour in non-clinical samples.

### Overview and predictions

**FIGURE 1:**
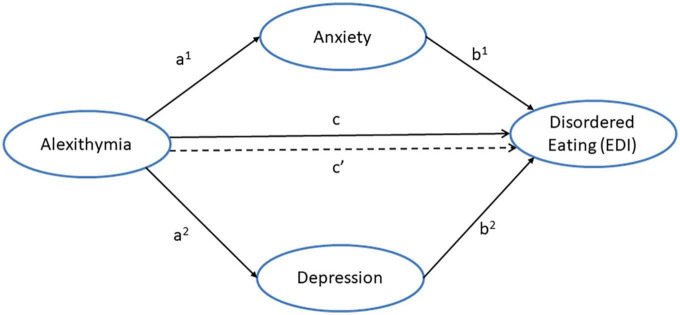
Proposed mediation model illustrating the direct pathway (c’) and indirect pathways between alexithymia and disordered eating *via* the dual mediators of anxiety (a^1^ + b^1^) and depression (a^2^ + b^2^).

The aims of the current study were to (i) confirm findings that disordered eating is positively related to alexithymia (DIF and DDF) in non-clinical participants, and (ii) to examine if depression and/or anxiety would mediate the influence of alexithymia on disordered eating in a non-clinical sample. Participants completed self-report measures of alexithymia (TAS-20), negative affect (depression and anxiety) and disordered eating (*drive for thinness, bulimia*, and *body dissatisfaction*). Mediation analyses were conducted to examine the potential role of negative affect in the relationship between alexithymia and disordered eating. The proposed mediation model is presented in Figure 1. Path (a) is the relationship between the predictor alexithymia subscale and the mediators (depression and anxiety). Path (b) is the relationship between the mediators and the dependent variable (disordered eating). The total effect (c) depicts the combined effects of alexithymia and negative affect on disordered eating. The direct effect (c’) illustrates the relationship between alexithymia and disordered eating, after controlling for the mediators (depression and anxiety), whereas the indirect effect (a + b) refers to the relationship between alexithymia and disordered eating *via* the mediators. Given that drive for thinness, bulimia, and body dissatisfaction are dimensional factors that cut across clinical and non-clinical populations (26, 27), the findings of direct and indirect effects of alexithymia on disordered eating, observed in clinically diagnosed patients (46, 47), were also expected in our non-clinical sample.

The predictions for the study are as follows: In line with previous findings (31, 34, 35), it was expected that *drive for thinness* would be positively related to alexithymia (DIF and DDF). However, given that controlling for negative affect can reduce the strength of the relationship between alexithymia and disordered eating (34), it was expected that depression and anxiety would mediate the relationship between alexithymia (DIF and DDF) and *drive for thinness*. Nevertheless, evidence that relationships between alexithymia and disordered are evident even after controlling for negative affect (29), suggest that a direct (independent) effect of alexithymia (DIF or DDF) on *drive for thinness* would still be evident.

In line with prior studies (31–35), it was expected that *bulimia* would be positively related to alexithymia (DIF and DDF). However, as controlling for negative affect can diminish the relationship between alexithymia and disordered eating (34), it was expected that depression and anxiety would mediate the relationship between DIF (or DDF) and *bulimia*. Nonetheless, as alexithymia can predict disordered eating independently of negative affect (29, 33), a direct effect of alexithymia (DIF or DDF) on *bulimia* was expected.

A positive relationship between *body dissatisfaction* and alexithymia (DIF and DDF) was expected, in line with previous work (29, 34). However, it was predicted that this relationship would be mediated by depression and anxiety, given that controlling for negative affect reduces the strength of relationship between alexithymia and disordered eating (34). Yet, as alexithymia predicts disordered eating, even after controlling for negative mood (29), it was expected that alexithymia (DIF or DDF) would also be an independent predictor of *body dissatisfaction.*

## Materials and methods

### Participants

Two-hundred and forty-eight female undergraduate students were recruited from the undergraduate populations of Aston University, Birmingham, UK and De Montfort University, Leicester, UK and took part for course credit. This was an opportunity sample where volunteers responded to electronic adverts on the universities’ research participant schemes. The inclusion criteria were healthy female students aged 18 or above. The exclusion criteria were males, as they differ from females in their expression of disordered eating (51, 52), and a history of depression and/or an eating disorder. The inclusion and exclusion criteria were included in the advert. No screening measure was used to determine clinical history; individuals with a history of depression and/or eating disorders were requested in the advert not to volunteer for the study. Examination of the participant characteristics (see Table 1) would tend to support that the current cohort represents a non-clinical sample. The mean age of the participants was 23.6 (SD = 6.2; range = 18 to 54). The ethnicities of the participants were not recorded, as we had no hypotheses concerning this factor. This sample size was chosen as n≈250 has been shown to result in reliable estimates of relationships between factors (53). It is also notable that the current sample is larger than the cohorts used in the related clinical mediation studies: *n* = 160 (46) and *n* = 72 (47). The study was approved by the research ethics committees of both institutions.

**TABLE 1:** Participant characteristics: mean values for age, mood, eating behaviour, and alexithymia (standard deviations are presented in parentheses).

*n* = 248	Mean (SD)	Range
Age	23.6 (6.3)	18–54
Alexithymia (TAS-20)	51.6 (15.3)	23–96
Difficulty describing feelings (DDF)	12.8 (4.3)	4–24
Difficulty identifying feelings (DIF)	15.7 (5.7)	7–35
Externally oriented thinking (EOT)	23.1 (9.56)	9–57
Eating disorders inventory (Total)	18.3 (15.5)	0–72
Drive for thinness (DFT)	5.31 (6.2)	0–21
Bulimia (BUL)	1.8 (3.3)	0–21
Body dissatisfaction (BD)	11.1 (8.7)	0–30
Anxiety (HADS)	7.13 (4.8)	0–20
Depression (HADS)	4.1 (3.8)	0–21

### Measures

The Toronto Alexithymia Scale [TAS-20; (6) is a 20-item self-report measure assessing three facets of alexithymia: *difficulty identifying feelings* (DIF; e.g., “I have feelings that I can’t quite identify”), *difficulty describing feelings* (DDF; e.g., “It is difficult for me to find the right words for my feelings”), and *externally oriented thinking* (EOT; e.g., “I prefer talking to people about their daily activities rather than their feelings”)]. Participants indicate the extent to which each item describes them using a 5-point Likert scale (1 = strongly disagree, 5 = strongly agree). Scores on the subscales range between 7–35 (DIF), 5–25 (DDF), and 8–40 (EOT) and are summed to give a total TAS-20 score, which ranges between 20 and 100, with higher scores indicating greater alexithymia. The TAS-20 has been shown to be valid (7) and reliable in student samples (54, 55). For example, recent published work by our group reported that scores on all three TAS-20 subscales was good: 0.70 for *difficulty identifying feelings*, 0.82 for *difficulty describing feelings*, and 0.71 for *externally oriented thinking* (56).

The Hospital Anxiety and Depression scale [HADS; (57)] is a 14-item measure of self-reported depression and anxiety. Each item consists of a statement relating to a symptom of depression (e.g., “I feel as if I am slowed down”) or anxiety (e.g., “I feel tense or wound up”) and participants indicate the frequency that they experience that symptom using a 4-point Likert scale. Scores for each item range from 0–3, and for each subscale between 0–21 with higher score equating to greater depression or anxiety. The HADS is a valid and reliable measure of depression and anxiety (58). This measure is also reliable in student samples, for example a recent study demonstrated that that both subscales were reliable: 0.68 for anxiety and 0.77 for depression (59).

The eating- and weight-related subscales from the Eating Disorders Inventory-II [EDI-II; (28)] were used to measure self-reported disordered eating: *drive for thinness* (DFT; e.g., “I am terrified of gaining weight”), *bulimia* (BUL; e.g., “I have gone on eating binges where I have felt that I could not stop”), and body-dissatisfaction (BD; e.g., “I think that my stomach is too big”). Participants rate the extent to which each statement is true of them using a 6-point Likert scale (ranging from *always* to *never*). All items are scored from 0–3 (from least to most severe disordered eating). Responses indicating the most severe symptoms are scored three, two, and one, respectively, whilst the remaining three responses are scored zero. Scores on the DFT and Bul subscales range between 0 and 21, and scores on the BD subscale range between 0 and 27. These scales have been shown to have good reliability in student samples (54). For example, recent published work by our group reported that the three eating- and weight-related subscales EDI all showed very good reliability: 0.89 for *drive for thinness*, 0.78 for *bulimia*, and 0.91 for *body dissatisfaction* (60).

### Procedure

Volunteers attended individual appointments in a private room within the psychology laboratories at the two universities. Having given full written informed consent, participants completed printed copies of the HADS, TAS-20, and EDI-II in a counterbalanced order.

### Data analysis

Data were analysed using Jamovi (version 2; The Jamovi Project, 2021). All data were treated as continuous variables. Spearman correlations were used to determine the significance of the associations between variables (as Shapiro-Wilks tests confirmed that none of the factors were normally distributed). Preliminary analysis revealed that difficulty identifying feelings and difficulty describing feelings subscales, but not the externally oriented thinking subscale, of the TAS-20 were correlated with all measures of disordered eating. Furthermore, DIF and DDF were strongly correlated (0.67) with each other, suggesting they were measuring similar constructs. Partial correlations revealed that, after controlling for DDF, DIF was still significantly associated with DFT and bulimia, but not body dissatisfaction. On the other hand, after controlling for DIF, DDF was only significantly correlated with body dissatisfaction and not DFT or bulimia. Therefore, DIF was included as the predictor in the mediation analyses for DFT and bulimia and DDF was included as the predictor in the mediation analysis for body dissatisfaction. Separate mediation analyses were conducted for each of the three eating subscales with depression and anxiety entered as parallel mediators, in line with previous research (47). The jMM module in Jamovi was used to conduct the mediation analyses. This procedure involved bootstrapping (5,000 iterations) to generate bias-corrected 95% confidence intervals (CI) for each effect. This method has been shown to effectively control for Type 1 errors (61).

## Results

### Participant characteristics

Comparison of the mean EDI scores from the current study (presented in Table 1) with the norms reported in a large non-clinical student sample (62) revealed that our participants scored significantly higher on all EDI subscales: DFT (mean difference = 1.6), t(248) = 4.12, *p* < 0.001; BUL (mean difference = 0.41), t(248) = 1.94, *p* = 0.053; and BD (mean difference = 1.29), t(248) = 2.33, *p* < 0.05. However, importantly, comparison of the current scores with the norms for a clinical sample (63) revealed that the current scores were significantly lower on all subscales of the EDI: DFT (mean difference = −9.1), t(248) = 22.9, *p* < 0.001; BUL (mean difference = −5.1), t(248) = 22.4, *p* < 0.001, and BD (mean difference = −7.38), t(248) = 13.3, *p* < 0.001, which supports the non-clinical status of the current sample.

Comparison of the mean TAS-20 total score with a recent UK student sample (54) revealed that our sample scored significantly higher, mean difference = 6.14, t(247) = 6.32, *p* < 0.001. However, importantly, the current TAS-20 score is significantly lower (mean difference = −12.2) than that reported in a sample of clinically diagnosed patients with eating disorders (46); t(205) = 12.5, *p* < 0.001. Comparison of depression and anxiety scores from the current study with a large UK sample of undergraduate students (64) revealed that the current sample was significantly less anxious (mean difference = −1.63) than the previous sample, t(247) = 5.34, *p* < 0.001. However, the current participants were slightly more depressed (mean difference = 0.73), t(246) = 3.01, *p* < 0.01. Importantly, in comparison to a clinical sample (65) the participants in the current were significantly less anxious (mean difference = −6.47, t(246) = 21.2; *p* < 0.001) and depressed (mean difference = −9.5, t(246) = 39.4, *p* < 0.001. Taken together the pattern is consistent with our conception of the current sample as non-clinical.

### Relationships between age, mood, alexithymia, and disordered eating

Correlational analyses (see Table 2) using Spearman’s tests revealed that disordered eating (*drive for thinness*, *bulimia*, and *body dissatisfaction*) was significantly positively related to alexithymia (DDF and DIF subscale scores, but not EOT) and mood (depression and anxiety). There were also significant associations between alexithymia and mood. It is notable that age was significantly related to alexithymia, disordered eating, and mood^1^. As observed relationships were based on Spearman correlations the mediational analyses were conducted using the ranked data.

**TABLE 2:** Spearman correlation coefficients for the relationships between age, mood, alexithymia, and eating behaviour.

Variable	1	2	3	4	5	6	7	8	9
Age	1	−0.17**	−0.24***	−0.23***	0.004	0.08	0.13	**−**0.09	0.03
DDF		1	0.67***	0.43***	0.35***	0.14**	0.29***	0.25***	0.38***
DIF			1	0.32***	0.41***	0.27***	0.27***	0.48***	0.44***
EOT				1	0.08	**−**0.11	0.12	−0.14*	0.19**
DFT					1	0.41***	0.65***	0.26***	0.36***
BUL						1	0.29***	0.35***	0.27***
BD							1	0.30***	0.36***
ANX								1	0.47***
DEP									1

TAS-20, Toronto Alexithymia Scale; DDF, difficulty describing feelings (TAS-20); DIF, difficulty identifying feelings (TAS-20); EOT, externally oriented thinking (TAS-20); EDI, Eating Disorders Inventory; DFT, drive for thinness (EDI); BUL, Bulimia (EDI); BD, body dissatisfaction (EDI); ANX, Hospital Anxiety and Depression Scale–anxiety subscale, and DEP, Hospital Anxiety and Depression Scale–depression subscale. *p < 0.05; **p < 0.01; ***p < 0.001. Bold values indicate significant relationships.

### The influence of alexithymia (DIF) and negative affect (depression and anxiety) on drive for thinness

**FIGURE 2:**
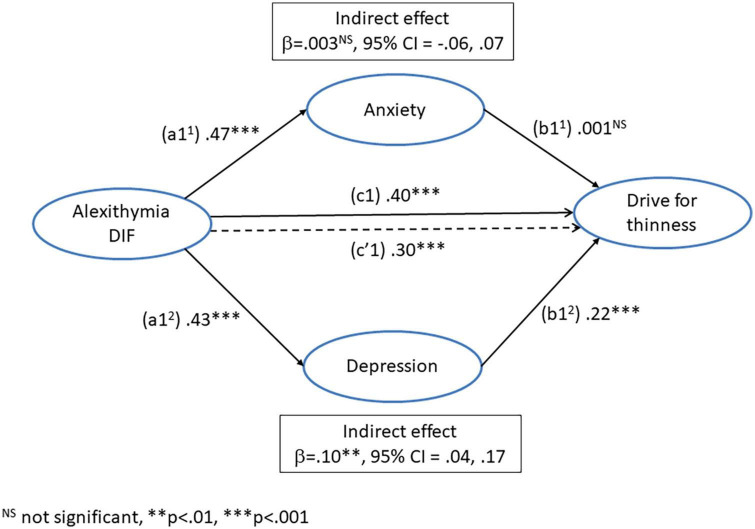
Mediation model for the direct (c1’) and indirect pathways between alexithymia (difficulty identifying feelings) and drive for thinness *via* the dual mediators of anxiety (a1^1^ + b1^1^) and depression (a1^2^ + b1^2^).

A regression was conducted with *difficulty identifying feelings* as the predictor variable, *drive for thinness* as the dependent variable, and anxiety and depression as parallel mediators. The paths of this model are illustrated in Figure 2 and the coefficients and 95% CI from this analysis are presented in Table 3.

**TABLE 3:** Path coefficients and 95% confidence intervals (estimated using bias corrected bootstrapping) for the mediation analyses of *difficulty identifying feelings* (DIF) on drive for thinness *via* the parallel mediators of depression and anxiety.

	Path estimates	Coefficient (SE)	LLCI	ULCI
Drive for Thinness	a1^1^	0.47 (0.05)***	0.37	0.57
	a1^2^	0.43 (0.06)***	0.32	0.54
	b1^1^	0.001 (0.07)^NS^	–0.12	0.14
	b1^2^	0.22 (0.07)***	0.10	0.35
	c1	0.40 (0.06)***	0.29	0.51
	c’1	0.30 (0.06)***	0.18	0.42

**Indirect effects**	**Path**	**Effect (SE)**	**LLCI**	**ULCI**

Difficulties identifying	Anxiety	0.003 (0.03)^NS^	–0.06	0.07
feelings (DIF)	Depression	0.10 (0.03)**	0.04	0.17

**p < 0.01, ***p < 0.001, ^NS^ = *p* > 0.05.

The path (a1^1^) from DIF to anxiety was significant, as was the path (a1^2^) from DIF to depression. The path (b1^1^) from anxiety to drive for thinness was not significant, but the path (b1^2^) from depression to drive for thinness was significant. The total effect of DIF on drive for thinness (c1) was significant, as was the direct effect (c’1), controlling for the influence of depression and anxiety. The indirect effect of DIF on drive for thinness (a1^1^ + b1^1^) *via* anxiety was not significant. However, the indirect effect *via* depression (a1^2^ + b1^2^) was significant. This suggests that the influence of DIF on *drive for thinness* was mediated by depression but not anxiety. However, even after controlling for negative affect, DIF remained an independent predictor of *drive for thinness.*

### The influence of alexithymia (DIF) and negative affect (depression and anxiety) on *bulimia*

**FIGURE 3:**
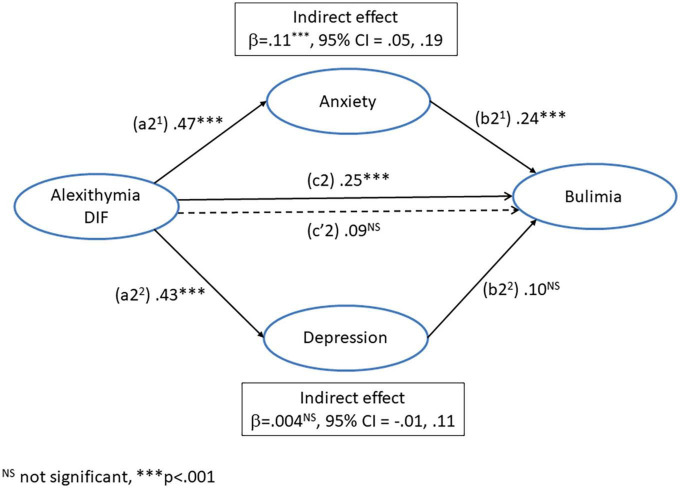
Mediation model for the direct (c2’) and indirect pathways between alexithymia (difficulty identifying feelings) and bulimia *via* the dual mediators of anxiety (a2^1^ + b2^1^) and depression (a2^2^ + b2^2^).

A regression was conducted with *difficulty identifying feelings* as the predictor variable, *bulimia* as the dependent variable, and anxiety and depression as parallel mediators. The paths of this model are illustrated in Figure 3 and the coefficients and 95% CI from this analysis are presented in Table 4.

**TABLE 4:** Path coefficients and 95% confidence intervals (estimated using bias corrected bootstrapping) for the mediation analyses of *difficulty identifying feelings* (DIF) on bulimia via the parallel mediators of depression and anxiety.

	Path estimates	Coefficient (SE)	LLCI	ULCI
Bulimia	a2^1^	0.47 (0.05)***	0.37	0.57
	a2^2^	0.43 (0.06)***	0.32	0.54
	b2^1^	0.24 (0.06)***	–0.12	0.14
	b2^2^	0.10 (0.06)^NS^	–0.02	0.23
	c2	0.25 (0.06)***	0.13	0.36
	c’2	0.09 (0.07)^NS^	–0.05	0.22

**Indirect effects**	**Path**	**Effect (SE)**	**LLCI**	**ULCI**

Difficulties identifying	Anxiety	0.11 (0.03)***	0.05	0.19
feelings (DIF)	Depression	0.04 (0.03)^NS^	**−**0.01	0.11

***p < 0.001, ^NS^ = *p* > 0.05.

The path (a2^1^) from DIF to anxiety was significant, as was the path (a2^2^) from DIF to depression. The path (b2^1^) from anxiety to *bulimia* was significant, but the path (b2^2^) from depression to *bulimia* was not significant. The total effect of DIF on *bulimia* (c2) was significant, but the direct effect (c’2) was no longer significant, after controlling for the influence of depression and anxiety. The indirect effect of DIF on bulimia (a2^1^ + b2^1^) *via* the mediator (anxiety) was significant. However, the indirect effect *via* depression (a2^2^ + b2^2^) was not significant. This suggests that the influence of DIF on *bulimia* was mediated by anxiety but not depression. Furthermore, after controlling for negative affect DIF no longer directly predicted *bulimia.*

### The influence of alexithymia (DDF) and negative affect (depression and anxiety) on *body dissatisfaction*

**FIGURE 4:**
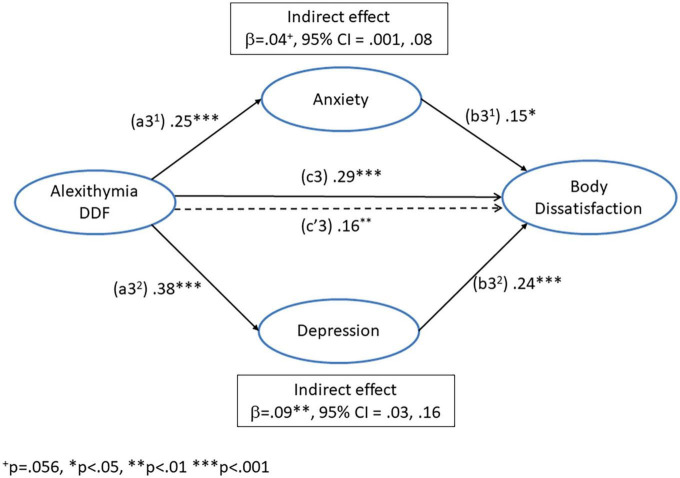
Mediation model for the direct (c3’) and indirect pathways between alexithymia (difficulty describing feelings) and body dissatisfaction *via* the dual mediators of anxiety (a3^1^ + b3^1^) and depression (a3^2^ + b3^2^).

A regression was conducted with *difficulty describing feelings* as the predictor variable, *body dissatisfaction* as the dependent variable, and anxiety and depression as parallel mediators. The paths of this model are illustrated in Figure 4 and the coefficients and 95% CI from this analysis are presented in Table 5.

**TABLE 5:** Path coefficients and 95% confidence intervals (estimated using bias corrected bootstrapping) for the mediation analyses of *difficulty describing feelings* (DDF) on body dissatisfaction *via* the parallel mediators of depression and anxiety.

	Path estimates	Coefficient (SE)	LLCI	ULCI
Body dissatisfaction	a3^1^	0.25 (0.06)***	0.12	0.37
	a3^2^	0.38 (0.06)***	0.27	0.48
	b3^1^	0.15 (0.07)*	0.01	0.27
	b3^2^	0.24 (0.07)***	0.10	0.38
	c3	0.29 (0.06)***	0.17	0.41
	c’3	0.16 (0.07)**	0.02	0.29

**Indirect effects**	**Path**	**Effect (SE)**	**LLCI**	**ULCI**

Difficulties describing	Anxiety	0.04 (0.02)^+^	0.001	0.08
feelings (DDF)	Depression	0.09 (0.03)**	0.03	0.16

^+^*p* = 0.056, **p < 0.01, ***p < 0.001. ^1^All mediations were recalculated with age entered as a covariate, but as the results did not change, we report the findings without this covariate for ease of understanding.

The path (a3^1^) from DDF to anxiety was significant, as was the path (a3^2^) from DDF to depression. The path (b3^1^) from anxiety to *body dissatisfaction* was significant, as was the path (b3^2^) from depression to *body dissatisfaction*. The total effect of DDF on *body dissatisfaction* (c3) was significant, as was the direct effect (c’3), after controlling for the influence of depression and anxiety. The indirect effect *via* depression (a3^2^ + b3^2^) was significant. However, the indirect effect of DDF on *body dissatisfaction* (a3^1^ + b3^1^) *via* anxiety was only trend significant (*p* = 0.056). This suggests that the influence of DDF on *body dissatisfaction* was mediated by depression and to some extent anxiety. Furthermore, after controlling for negative affect, DDF remained an independent predictor of *body dissatisfaction.*

## Discussion

The aims of the current study were to (i) confirm findings that disordered eating is positively related to alexithymia (DIF and DDF) in healthy participants, and (ii) to examine if depression and/or anxiety would mediate the influence of alexithymia on disordered eating in a non-clinical sample. The expectation that DIF and DDF would be positively related to *drive for thinness* (DFT) was supported by the current findings. This is consistent with previous studies (31, 33, 35). The prediction that depression and anxiety would mediate the influence of alexithymia on *drive for thinness* was partially supported, as depression but not anxiety mediated the link between DIF and DFT. As expected, DIF remained as an independent predictor of *drive for thinness*, after controlling for negative affect. This is consistent with previous work in healthy participants (29). The findings in our non-clinical sample of direct and indirect effects of alexithymia on eating behaviour (*drive for thinness*) *via* depression corresponds with evidence from clinical samples (46, 47).

The prediction that DIF and DDF would be positively correlated with *bulimia* was supported by the current findings. This is consistent with previous studies (31–35). The expectation that depression and anxiety would mediate the influence of alexithymia on *bulimia* was partially supported, as anxiety but not depression mediated the influence of DIF on *bulimia*. The prediction that DIF would be an independent predictor of *bulimia*, after depression and anxiety had been controlled, was also not supported. This is consistent with previous findings that negative affect accounted for the relationship between alexithymia and *bulimia* (34). However, it does not correspond with evidence that alexithymia is an independent predictor of bulimia (33). The current evidence that anxiety mediated the relationship between DIF and *bulimia* is consistent with previous work in clinical patients (47), but the finding that depression did not mediate this relationship does not correspond with the results of that study.

The prediction that *body dissatisfaction* would be positively related to DIF and DDF was supported by the current findings. This is consistent with the evidence from previous studies (29, 34). The expectation that the link between alexithymia and body dissatisfaction would be mediated by depression and anxiety was supported by the current findings, although the mediation by anxiety was only trend significant (*p* = 0.056). This is consistent with evidence that negative affect accounts for the relationship between alexithymia and disordered eating (34) and with studies in clinical participants (46, 47). Furthermore, the prediction that alexithymia (DDF) would remain as an independent predictor of *body dissatisfaction* after controlling for the influence of negative affect was supported by the current data. This is consistent with evidence that alexithymia is an independent predictor of disordered eating, after controlling for negative affect (29) and with the findings in clinical samples (46, 47).

Alexithymia is considered a relatively stable trait (24, 25) that is a risk factor for depression (21, 22) and anxiety (23), which in turn are risk factors for disordered eating (41, 43, 44). Thus, one explanation for the current findings is that alexithymia led to increases in negative affect, which then led to disordered eating. Consistent with this line of thought, the proposed explanation for a previous finding that negative affect mediated the influence of interpersonal problems on binge eating, was that interpersonal problems led to higher negative affect, which in turn led to increased tendency to binge eating (66). Longitudinal research is required to confirm the direction of this predictive model.

There are a number of limitations to the current study that need to be considered. Firstly, we did not conduct reliability analyses on the questionnaire data. However, these measures have been shown to be reliable in previous student samples, including in studies published by our group (56, 59, 60). Some of the current findings, i.e., the mediation of *drive for thinness* by anxiety and the mediation of *bulimia* by depression were very small effects. Although our sample (*n* = 248) was larger than the cohorts used in related the clinical mediation studies (46, 47) and similar sample sizes have been shown to lead to stable correlations (53), our study was not powered to detect these very small effects. Nevertheless, *a posteriori* power analysis based on published sample sizes required for mediation (67) confirm that our study was powered to detect small to medium effect sizes, thus the remaining findings were sufficiently powered. Another issue to consider is the measure of alexithymia that was used in the current study. In line with previous clinical mediation studies (46, 47) we used the TAS-20 to measure alexithymia, thus the findings might not generalise to other measures of alexithymia [e.g., Bermond–Vorst Alexithymia Questionnaire; BVAQ; (68)]. Nevertheless, scores on these measures tend to be significantly correlated. For example, it has been reported (69) that total TAS-20 and BVAQ scores were positively related (*r* = 0.62), with the subscales measuring difficulty identifying and describing feelings correlating even more strongly (*r* = 0.7 and *r* = 0.8, respectively). This suggests that similar findings would have been observed had the BVAQ been used in place of the TAS-20. However, it is notable that self-report measures of emotion processing deficits like the TAS-20 and BVAQ do not always correlate with implicit measures of emotion processing. For example, a recent study showed that participants with obesity reported similar levels of alexithymia to normal weight controls, but demonstrated deficits on an implicit emotion processing task (70). With this in mind, incorporating implicit measures of emotional awareness into future studies as well as self-report questionnaires might improve the predictive power of the model relating alexithymia, negative affect, and disordered eating. Finally, it is notable that the current sample consisted of females only. Future work is needed to examine if alexithymia predicts disordered eating in males and if this relationship is mediated by negative affect.

In sum, the findings of the current study confirmed that depression mediated the influence of alexithymia on *drive for thinness* and *body dissatisfaction* but not *bulimia*. On the other hand, anxiety mediated the influence of alexithymia on *bulimia* and *body dissatisfaction*, but not *drive for thinness.* It is possible that depression might mediate *bulimia* and anxiety *drive for thinness*, but the size of these mediation effects was too small to be detected by the current sample. Although negative affect mediated the influence of alexithymia on all measures of disordered eating, alexithymia (DIF and DDF, respectively) was still an independent predictor of *drive for thinness* and *body dissatisfaction*. One implication of our work is that future studies examining the potential roles of alexithymia and negative affect in the development of disordered eating should aim for larger sample sizes (*n*≈400) to ensure that they are also powered to detect very small effects. Another implication is that the influence of alexithymia on disordered eating (at least *drive for thinness* and *body dissatisfaction*) cannot be entirely explained by negative affect. The current findings are cross-sectional and correlational and, thus, cannot provide evidence of the direction of causation. However, as alexithymia is considered to be a relatively stable trait that is a risk factor for disordered eating (15, 16), targeted interventions to address deficits in recognising and describing ones feelings could potentially be beneficial for individuals who are “at risk” of developing eating disorders. This is potentially important, as early intervention has been shown to be the key in treating and preventing eating disorders (71).

## Data availability statement

The datasets presented in this study can be found in online repositories. The names of the repository/repositories and accession number(s) can be found below: The data is available *via* Mendeley data DOI: 10.17632/tfbh8vsrkz.3.

## Ethics statement

The studies involving human participants were reviewed and approved by the Research Ethics Committees of both Aston University and De Montfort University. The patients/participants provided their written informed consent to participate in this study.

## Author contributions

NR and DW designed and supervised the study and wrote the manuscript. Data was collected by undergraduate research assistants. NR analysed the data. Both authors contributed to the article and approved the submitted version.
